# Breaking the Waves: Modelling the Potential Impact of Public Health Measures to Defer the Epidemic Peak of Novel Influenza A/H1N1

**DOI:** 10.1371/journal.pone.0008356

**Published:** 2009-12-21

**Authors:** Matthias an der Heiden, Udo Buchholz, Gérard Krause, Göran Kirchner, Hermann Claus, Walter H. Haas

**Affiliations:** Department of Infectious Disease Epidemiology, Robert Koch Institute, Berlin, Germany; University of British Columbia, Canada

## Abstract

**Background:**

On June 11, 2009, the World Health Organization declared phase 6 of the novel influenza A/H1N1 pandemic. Although by the end of September 2009, the novel virus had been reported from all continents, the impact in most countries of the northern hemisphere has been limited. The return of the virus in a second wave would encounter populations that are still nonimmune and not vaccinated yet. We modelled the effect of control strategies to reduce the spread with the goal to defer the epidemic wave in a country where it is detected in a very early stage.

**Methodology/Principal Findings:**

We constructed a deterministic SEIR model using the age distribution and size of the population of Germany based on the observed number of imported cases and the early findings for the epidemiologic characteristics described by Fraser (Science, 2009). We propose a two-step control strategy with an initial effort to trace, quarantine, and selectively give prophylactic treatment to contacts of the first 100 to 500 cases. In the second step, the same measures are focused on the households of the next 5,000 to 10,000 cases. As a result, the peak of the epidemic could be delayed up to 7.6 weeks if up to 30% of cases are detected. However, the cumulative attack rates would not change. Necessary doses of antivirals would be less than the number of treatment courses for 0.1% of the population. In a sensitivity analysis, both case detection rate and the variation of R0 have major effects on the resulting delay.

**Conclusions/Significance:**

Control strategies that reduce the spread of the disease during the early phase of a pandemic wave may lead to a substantial delay of the epidemic. Since prophylactic treatment is only offered to the contacts of the first 10,000 cases, the amount of antivirals needed is still very limited.

## Introduction

On June 11, 2009, the World Health Organization (WHO) declared Pandemic Phase 6 based on sustained community transmission in more than one WHO region [1]. By the end of September 2009, in most European countries the impact has been limited, which is likely due to the dampening effect of the summer months. What the future holds is difficult to predict as past pandemics have been rather variable because of the complex interactions of immunity, pathogenicity, season and other factors [2]. Many countries have established a stockpile of antivirals for the treatment of the (severely) sick. We examine how a control strategy in the very beginning could defer the peak of the epidemic by using only a very small part of the stored antivirals for prophylactic treatment.

There are a number of possible public health measures that may be used to stop or slow down the spread. Border control has been extensively discussed in this context to delay the international spread of influenza. However, in order to achieve a significant delay, more than 99% of air travel would have to be stopped [3]. As has been shown for SARS, entry screening methods are unlikely to detect more than 10% of imported infections and the positive predictive value of temperature screening is low especially at the beginning of a pandemic [4], [5]. It is therefore inevitable that importation occurs. For the management of imported cases, other measures may be used and include contact tracing, isolation and quarantine, as well as post exposure prophylaxis. It is unclear; however, which public health strategy could be effective in preventing the spill-over from imported cases and slow-down the transmission within the general population. Even less clear is how long these measures should be maintained, particularly once that domestic transmission has started.

Mathematical models can be used to aid in decision making and have been increasingly applied to analyse the potential impact of containment strategies, pharmaceutical interventions and public health measures on the course of a novel influenza pandemic [6]–[9]. Before the emergence of the novel influenza A/H1N1, virus modelling studies have suffered from the high number and uncertainty of necessary assumptions [10]. Even during the first months of the pandemic with the novel virus A/H1N1 only limited knowledge has emerged about the characteristics of the new virus [11], [12]. Therefore, we do not stress the particular timing and severity of a certain baseline scenario, but rather concentrate on the effect of the intervention in the model that should allow an estimation of the possible effects of these interventions.

To construct possible baseline scenarios we used some of the estimates of properties of the novel virus that have been published based on data from the initial outbreak in Mexico by Fraser [11]. Similar estimates for R0 and a little higher estimates for the generation time were found in the USA [13]. We constructed a deterministic model with the following goals:

1. To model a possible evolution of an epidemic in Germany including assumptions about importation and domestic spread using the present knowledge about the virus;
2. to quantify the potential impact of public health measures, such as case detection, case isolation, quarantine of contacts, and the use of antiviral medication for therapy and post exposure prophylaxis, with a given effectiveness, on the initial evolution of the epidemic;
3. to identify possible conditions, which – if known – favour the adaptation of measures or the termination of the control strategies.

## Results

### Number of Imported Cases

Until June 5th, the rate of imported cases has been stable in Germany. The first identified cases in Germany were confirmed on April 29^th^, and until June 5^th^ a total number of 49 confirmed cases had been reported (Figure S1(A)). Of these 41 (84%) had a known travel history to Mexico, the United Kingdom or the USA; of the remaining 8 cases 7 had been contacts to one of the imported cases and for one case the source case was unknown but might have been related to contacts with travellers (airport worker). The average number of imported cases during this time period (38 days) corresponds to 1.1 cases per day, however, 28 of the 41 imported cases were reported during the 10 days prior to June 5^th^ (corresponding to 2.8 imported cases per day). We considered two different scenarios of case importation (Figure S1(B)): firstly, a constant number of imported cases per day (five or ten cases per day), and secondly, an exponentially growing number of imported cases limited to 120 imported cases per day. The exponential growth rate was determined by assuming an “import-R0” of 1.1 and the same generation time as for the transmission inside Germany.

### Model of Possible Evolution of an Epidemic in Germany

An example for the prevalence of symptomatic cases resulting from our SEIR model is shown in Figure S2 for three different values of R0 (1.34, 1.58, and 2.04). For an R0 of 1.58 the point estimate of the timing of the peak after introduction of the first case would be 10 to 11 weeks (depending on the number of imported cases, compare Table 1), the point estimate for the peak prevalence of the population infected would be 4.3%, for the total attack rate of the population infected 44.8% and for the population diseased 38.5% (Table 1). Depending on the three R0 the cumulative proportions of children that develop symptoms are 48%, 67%, and 79%, and the cumulative proportion of symptomatic adults 17%, 34%, and 54%, respectively. As a result of the higher susceptibility of children in the model the peak proportion of infected children is reached 18, 12 or 8 weeks (126, 82, or 56 days) after the first infected case, roughly 1-1.5 weeks earlier (i.e. 11, 8 or 6 days) than in adults (data not shown).

**Table 1:** Characteristics of the baseline scenario without preventive interventions under the assumption that each day 5 cases were imported to Germany.

R0		peak time (in weeks)	attack rate (in %)	peak prevalence (in %)	duration above 1% (in weeks)	duration above 0.1% (in weeks)
1.34	total infected	14.9	27.1	1.7	4.4	12
	symptomatic	14.9	23.3	1.5	3.7	11.6
1.58	total infected	10.3	44.8	4.3	5.2	9.6
	symptomatic	10.3	38.5	3.7	4.8	9.3
2.04	total infected	7	67.6	10.2	4.3	6.9
	symptomatic	7	58.1	8.8	4.1	6.7

The other importation scenarios (10 cases per day, exponentially growing number per day) lead to very similar characteristics.

### Model of the Impact of Public Health Measures

Figure S3 shows how the peak is delayed for an assumed R0 of 1.58 and 5 imported cases per day when the first 500 cases are managed with a combination of intensive case-based measures (CCM1), followed by 10,000 with management mainly restricted to members of the household (CCM2; for details see Methods section). The effect of the number of household focused interventions (CCM2) on the delay of the peak is dependant on the basic reproduction number R0 and the sensitivity of the surveillance system (Figure S4). When R0 is at least 1.58 and not more than 30% of cases can be detected, saturation occurs relatively early. Even if 50% of the cases can be detected or R0 is as small as 1.34 the peak of the epidemic can not be deferred any more after management of approximately 10,000 cases with CCM2. In general can be said: the higher R0 the earlier management with CCM2 becomes ineffective. This can only be balanced to a certain degree by a higher sensitivity of the surveillance system.

The following considerations are done on the basis of 5 imported cases per day and R0 equal to 1.58.

#### Effect of sensitivity of the surveillance system

The delay of the peak increases with the proportion of detected cases. When 10% of cases are detected and these are followed-up with CCM1 for the initial 500 cases (but no CCM2) the delay is 6 days, but can be raised to 20 days (gain of 2 weeks) when case detection is improved to 30%. The combined approach of 500 cases targeted with CCM1 and additional 10,000 cases with CCM2 the gain based on the improved case detection results in an increase for up to 6 weeks (11 to 50 days; Figure S3, Table 2).

**Table 2:** Delay (in days) of the peak of the epidemic wave for adults in Germany as a function of the number of imported cases (column A), the number of CCM1 and CCM2 treatments, R0 and the case detection rate (10%, 30% or 50%).

			CCM2 for following								
			0 cases			5,000 cases			10,000 cases		
A	CCM1	Ro	Case detection rate			Case detection rate			Case detection rate		
			10%	30%	50%	10%	30%	50%	10%	30%	50%
5 imported cases/day	for first 100	1.34	7	15	19	21	164	>180	24	>180	>180
		1.58	4	10	16	10	42	>180	11	49	>180
		2.04	3	7	11	5	17	48	5	18	56
	for first 500	1.34	12	40	74	22	172	>180	24	>180	>180
		1.58	6	20	47	10	43	>180	11	50	>180
		2.04	3	10	22	5	17	49	5	18	57
10 imported cases/day	for first 100	1.34	5	9	11	18	109	>180	21	163	>180
		1.58	3	7	10	9	35	180	10	42	>180
		2.04	1	4	7	4	14	39	4	16	47
	for first 500	1.34	9	26	40	18	114	>180	21	167	>180
		1.58	5	15	28	9	36	194	10	42	>180
		2.04	2	8	15	4	14	40	4	16	47
increasing number of cases/day (R0 = 1.1)	for first 100	1.34	9	18	23	22	72	94	25	89	126
		1.58	5	15	23	11	45	90	12	51	111
		2.04	3	9	17	5	19	53	5	21	61
	for first 500	1.34	13	34	42	22	74	97	25	90	129
		1.58	7	25	44	11	46	92	12	52	113
		2.04	3	12	29	5	19	54	5	21	61

#### Separate analysis of the effect of CCM1 and CCM2

In the example above 55% (6 of 11 days; 10% case detection rate) or 40% (20 of 50 days; 30% case detection rate) of the delay, respectively, is already achieved through CCM1 alone (Table 2). This holds similarly in the scenario of the exponentially growing number imported cases – of course under the assumption that already in the beginning the surveillance system detects a percentage of the imported cases.

#### Effect of R0

All of the above calculations are very sensitive to the value of R0. If R0 is as low as 1.34 it is much easier to delay the peak. If it is high, such as 2.0 or more, and the number of imported cases is at least 5 per day, then the effect of CCM1 and CCM2 can delay the peak at most by 8 weeks (57 days; Table 2), but only when case detection rate is high (50%).

### Estimation of the Resources Needed

The personnel and personnel time that is needed to implement such measures depends on the number of local health departments involved, their capacity and many other factors. The maximum number of antivirals, however, can be estimated. Assuming that each case has 15 contacts that merit attention for CCM1 and would get antiviral post exposure prophylaxis, this would result in a maximum of 500×15 ( = 7,500) treatment courses (as the number of doses for treatment equals the number of doses for prophylaxis). Further, if the household members of 10,000 cases are given antiviral prophylaxis this would amount to another 10,000×3 ( = 30,000) treatment courses, in total 37,500 treatment courses.

## Discussion

We present here a model how public health measures can contribute to the delay of an epidemic wave with the novel influenza virus A/H1N1 when the epidemic is detected in a very early stage. Delaying the pandemic spread is an important achievement because it gains time for other measures and preparations, such as early assessment of the virus' characteristics, activation of surge capacities or vaccine production and the development of a vaccination strategy [10].

The transmission parameters of our model were derived from the initial analysis of the epidemic in Mexico by Fraser [11] and a constant or slowly increasing influx of imported cases as observed for one month after the identification of the first case in Germany. This is also in agreement with European data showing a constant proportion of travel-related cases over time (the travel related cases within Europe between April 16 and June 2 also seem to remain constant over time [14]).

It has been shown before that so called targeted layered containment strategies, a combination of antiviral prophylaxis and non-pharmaceutical interventions, can be effective in reducing the transmission of pandemic influenza [8]. We extended this approach by analyzing the effect of a more intensive phase including contact tracing, identification and management of contacts outside of the household (CCM1), followed by household centred measures (CCM2).

The assumed effectiveness for CCM1 indicates that a corresponding strategy would be very effective in reducing the spread of the epidemic, if R0 is moderate or low and if the number of imported cases does not increase rapidly. In contrast, the household centred measures (CCM2) continue to gain time, even when larger amounts of cases are imported per day. In the model, the effect of CCM1 and CCM2 rapidly decreased for higher values of R0, indicating that this intervention should be stopped when the delay becomes only marginal. To our knowledge, the question of conditions that would lead to stopping interventions (or the lowered effects of specific measures) has so far been studied only in the context of community mitigation strategies and interventions, such as school closures [15].

Modelling studies analyzing preventive measures often assume that the intervention will be available for a large part or the whole population [6], [8], [16]. E.g. Carrat et al. analysed the effect of combined interventions targeting up to 70% of households in their “small-world-like” model [17]. In contrast, our model focuses on the initial stage and the first few thousand cases in Germany, and on the delay that can be achieved. We have restricted the analysis of maintaining the less intensive CCM2 strategy for 10,000 courses. The maximum amount of antivirals used in this approach corresponds to treatment courses for less than 0.1% of the German population and 5% of the amount available for seasonal influenza in the winter season. After the end of isolation, the treated contacts were assumed to remain susceptible to the infection. However, these conservative assumptions result only in a shift (depending on R0 significant) of the epidemic curve and virtually no reduction of the overall attack rate.

As we have shown, a number of factors are important when effects of public health interventions are considered. First, the rate of seeding has significant impact on the delay that can be achieved by the interventions. Other published models start their simulation with one infection, a random number each day or an increasing number at a reduced R0. In contrast, our analysis is based on the observed number of cases. Second, the sensitivity of the surveillance system to detect cases is important. In reality, detection of cases without travel history to countries with community transmission of novel influenza A/H1N1, i.e. domestic cases, may be difficult as the clinical picture of the disease has proven to be often non-specific. Therefore, sensitivity to detect cases may be relatively low. Given these limitations we have provided a number of different scenarios (case detection rates of 10, 30 and 50%) addressing how the described interventions may impact the epidemic.

As a note of caution it must be mentioned that these calculations should of course not be mistaken as a prediction. It was for example not possible to validate the assumptions about the effectiveness of the interventions using real data.

Other limitations are: (1) While before the start of the novel A/H1N1 epidemic it was difficult to make realistic assumptions, it is now easier to do so now, since first estimates can be drawn for a number of parameters of the novel influenza virus. Nevertheless, many pieces of information are uncertain and may change due to more information coming to light, or due to real changes of the virus and its epidemiology. (2) The effect of season is not taken into account, we expect that the virus is more easily transmitted in the fall or winter time [18], [19]. (3) The proportion of asymptomatic cases and their contribution to transmission is still unknown. (4) Lacking realistic alternative information we distinguished only two age groups, children and adults. (5) The evolution of the number of cases imported is unknown and will probably change over time. (6) The age dependence of susceptibility in Germany is unknown and is likely to differ from the one in Mexico. (7) The sensitivity of surveillance is unknown, and therefore the true proportion of cases detected is also unknown. If persons were infected in Germany from a source without travel history, then they may have been more easily missed, especially since initial surveillance efforts usually focus on diseased persons with travel history. (8) The rigor of public health measures is likely to vary among different local health departments. However, it is plausible that the measures taken contributed to the delay of the initial spread of the infection, because, until mid June at latest, virtually no tertiary cases had been detected by close surveillance of contacts and the surrounding of cases.

In the model the change from CCM1 to CCM2 was suggested on a population level. In reality, of course, there might not be a real threshold and the strategy might change depending on the individual resources of local health authorities. Of course with the change of the epidemiologic picture more rigorous measures of social distancing, such as school closures may be implemented. When leaving the intensive phase of contact tracing and case management (CCM1), we believe that CCM2, or a strategy with similar effect, might be well suited to follow after because it focuses on the household. This is a much more amenable unit and is based on the knowledge that being a member of a household with a confirmed case is the highest single risk factor for influenza infection [6].

In conclusion, despite the many possible pitfalls and caveats of our study we believe that we have demonstrated the possible impact of a sequential strategy on the spread of the novel influenza virus A/H1N1 in a country where imported cases start the epidemic.

## Materials and Methods

### Number of Imported Cases

Cased-based information was used to assign reported confirmed cases in Germany and status of either *imported* or *domestic*. Cases with travel history of more than 7 days before onset of symptoms (two times the maximal incubation period) were considered domestic.

### The Model

(a) Assumptions. We assumed that at the outset of the epidemic the entire population is fully susceptible to infection with the influenza A/H1N1 virus. Infectiousness was assumed to be equal in symptomatic and asymptomatic persons. This is based on the rationale that a lower degree of infectiousness is coupled with unrestricted mobility resulting in a higher number of potentially infectious contacts. In comparison, a higher degree of infectiousness in symptomatic patients is compensated by the reduction of the number of contacts, because patients are isolated and bedbound.

Assuming that the epidemiologic and virologic characteristics are similar to the epidemic in Mexico allowed us to use the values as described by Fraser [11]. They found the “most likely” basic reproductive number was 1.58, range 1.34 to 2.04, and estimated a generation time of 1.91 days (95% confidence interval 1.3–2.71). They distinguished children (<15 years of age) and adults (> = 15 years of age) and found that children were 2.06 (95% confidence interval 1.60–3.31) as susceptible as adults. The assortativity of mixing between children and adults was estimated as 0.5 (95% confidence interval 0.00–0.72) - an assortativity of 0 corresponds to a completely random mixing, whereas 1 corresponds to fully assortative groups. Finally, they found that 86% (95% confidence interval 69%–100%) of the infected persons become symptomatic. We considered three different scenarios of R0, namely R0 equal to 1.34, 1.58 or 2.04 and used the point estimates for all other parameters. The model does not incorporate assumptions about the severity of disease or how severity might alter infectiousness.

### Estimation of the Impact of Public Health Measures

Lastly we needed to make assumptions about the effectiveness of the public health measures and the sensitivity of the surveillance system. Assumptions are made for the effectiveness of two approaches that combined several case-based public health measures (combination of case-based measures; CCM):

1. Intensive measures, including isolation and therapy of cases, contact tracing, quarantine and post-exposure prophylaxis of selected contacts in- and outside of the household (CCM1). Because CCM1 consumes many resources it is assumed that CCM1 will only be sustained in the first phase. We model two scenarios with the first 100 or up to 500 cases cared for by CCM1 countrywide.
2. Less- intensive measures, focusing on the household, including isolation and therapy of cases, quarantine and post-exposure prophylaxis of household contacts (CCM2). Thus, we assume that following the initial 100–500 confirmed index cases there will be no contact tracing any more, i.e. no more post exposure prophylaxis for non-household contacts.

CCM1 and CCM2 are set to be 75% and 50% effective in reducing secondary cases, respectively. We modelled four scenarios: in the first and second, CCM1 is maintained for the first 100 cases followed by 5,000 or 10,000 cases cared for with a CCM2 strategy, in the third and forth, CCM1 is maintained for 500 cases followed by 5,000 and 10,000 cases with a CCM2 strategy, respectively.

To include the effect of surveillance we made assumptions about the number of imported cases that are recognized. For this purpose we varied the proportion of recognized imported cases from 0%, 10% and 30% to 50%. The assumed sensitivity of the surveillance system reflects the probability (10%, 30% or 50%) to detect domestic cases. A higher probability to detect imported cases would have led effectively to a reduced total number of imported cases per day in the model.

(b) Construction. We used – similar to Fraser [11] in their description of the outbreak in La Gloria – a generalised age-stratified deterministic SEIR model to describe the spread of the disease [20], [21].

We used the following assumptions about the age distribution and size of the population of Germany: 71,000,000 adult population (> = 15 years of age), 11,000,000 children (<15 years of age).

The complete model is described in the Appendix S1.

## Supporting Information

Figure S1 Confirmed imported (red) and domestic (blue) cases in Germany by date of onset of symptoms (A). The symptoms of the first confirmed case could be fixed for April 21. For three cases (one imported and two domestic cases), the date of onset of symptoms remained unknown; these cases have been assigned their reporting date (hatched boxes). (B) shows the three different modelled scenarios of importations.
(5.31 MB TIF)

Figure S2 Modelled evolution of the number of total (A) and age-stratified cases (B) with novel A/H1N1 virus in Germany. Parameters values are taken from Fraser (Science, 2007), and it is assumed that no preventive public health measures are taken. Prevalence of infectious cases is modeled for three values of Ro (1.31, 1.58, and 2.04) with the additional assumption that each day five cases were imported to Germany. The prevalence is calculated as proportion of infectious persons among the total population in the respective age group.
(4.72 MB TIF)

Figure S3 Delay of epidemic curve. The “most likely” Ro of 1.58 from the study of Fraser et al. (Science, 2007) was used and case detection rates of symptomatic cases were set to 10% and 30%, respectively. Ro is assumed to be 1.58, and each day five cases were imported. The household and non-household contacts of the first 500 detected cases were treated with a combination of case-based measures that include contact tracing, quarantine, and post-exposure prophylaxis (CCM1); and the household contacts of the next 10,000 cases were managed with strategy CCM2, which includes only preventive measures in the household of the cases.
(2.28 MB TIF)

Figure S4 Delay of the peak of the epidemic depending on the number of CCM2 treatments. The respective curves start where CCM1 has taken effect already in the first 500 detected cases. (A and B): The delay in days is presented for case detection rates of 10%, 30%, and 50%. R0 is set to 1.58. (A) shows the delay when 5 cases are imported per day, and (B) shows when an exponentially increasing number of cases, but not more than 120, are imported per day. (C and D): The delay in days is presented for basic reproduction numbers of 1.34, 1.58, and 2.04. The case detection rate is set to 30%. (C) shows the delay when 5 cases are imported per day, and (D) shows when an exponentially increasing number of cases, but not more than 120, are imported per day.
(7.72 MB TIF)

Appendix S1 Description of the SEIR model.
(0.01 MB TEX)
