# Supplementary figures and images for: Breaking the Waves: Modelling the Potential Impact of Public Health Measures to Defer the Epidemic Peak of Novel Influenza A/H1N1
Source: PLoS One. 2009 Dec 21;4(12):e8356. doi: 10.1371/journal.pone.0008356 (PMC2791869; doi:10.1371/journal.pone.0008356)

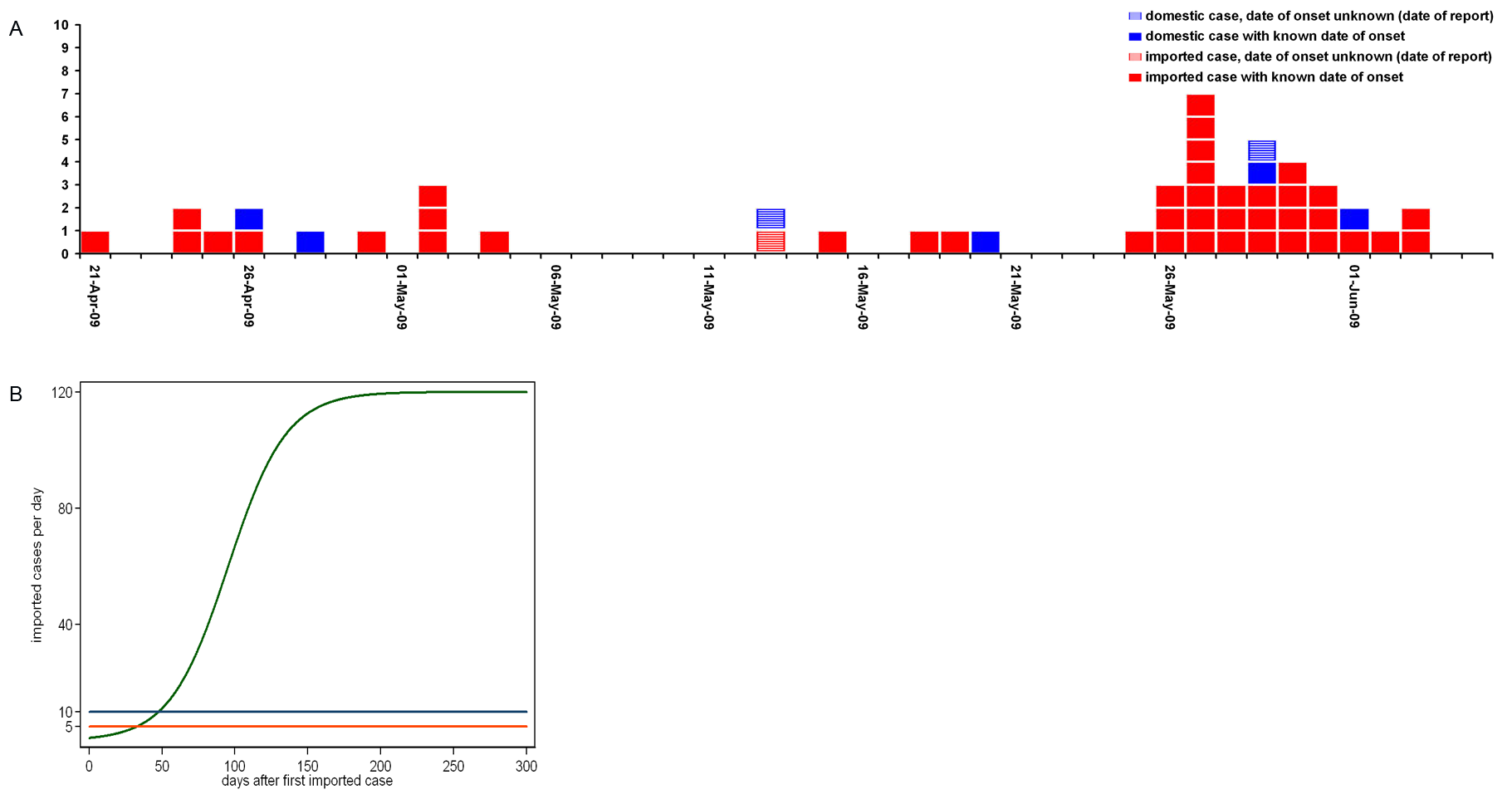

Supplement: Figure S1 — Confirmed imported (red) and domestic (blue) cases in Germany by date of onset of symptoms (A). The symptoms of the first confirmed case could be fixed for April 21. For three cases (one imported and two domestic cases), the date of onset of symptoms remained unknown; these cases have been assigned their reporting date (hatched boxes). (B) shows the three different modelled scenarios of importations. (5.31 MB TIF) [file pone.0008356.s001.tif]

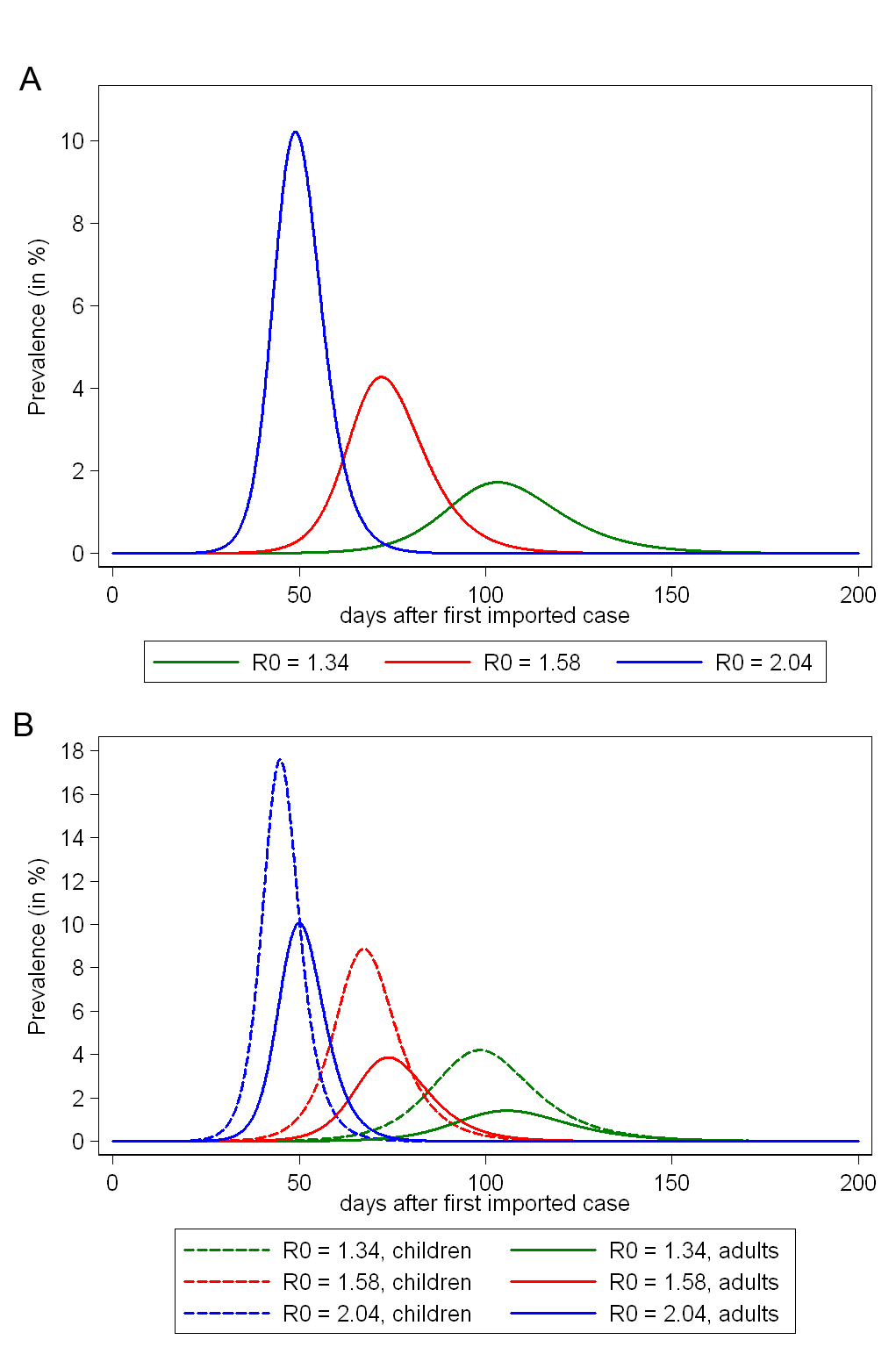

Supplement: Figure S2 — Modelled evolution of the number of total (A) and age-stratified cases (B) with novel A/H1N1 virus in Germany. Parameters values are taken from Fraser (Science, 2007), and it is assumed that no preventive public health measures are taken. Prevalence of infectious cases is modeled for three values of Ro (1.31, 1.58, and 2.04) with the additional assumption that each day five cases were imported to Germany. The prevalence is calculated as proportion of infectious persons among the total population in the respective age group. (4.72 MB TIF) [file pone.0008356.s002.tif]

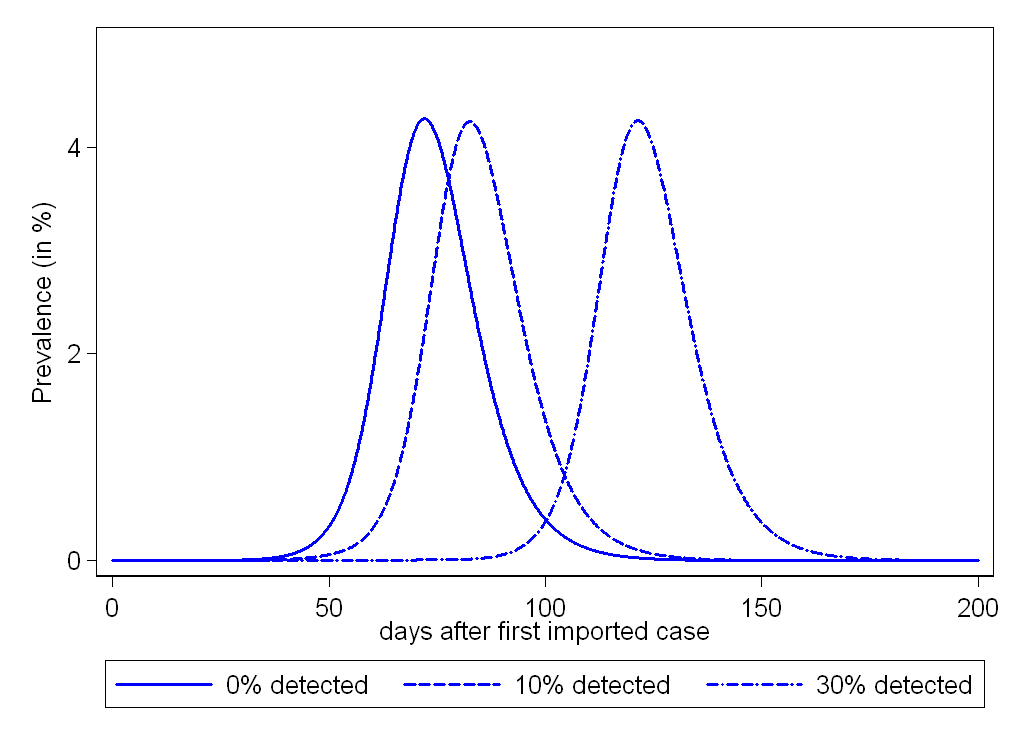

Supplement: Figure S3 — Delay of epidemic curve. The “most likely” Ro of 1.58 from the study of Fraser et al. (Science, 2007) was used and case detection rates of symptomatic cases were set to 10% and 30%, respectively. Ro is assumed to be 1.58, and each day five cases were imported. The household and non-household contacts of the first 500 detected cases were treated with a combination of case-based measures that include contact tracing, quarantine, and post-exposure prophylaxis (CCM1); and the household contacts of the next 10,000 cases were managed with strategy CCM2, which includes only preventive measures in the household of the cases. (2.28 MB TIF) [file pone.0008356.s003.tif]

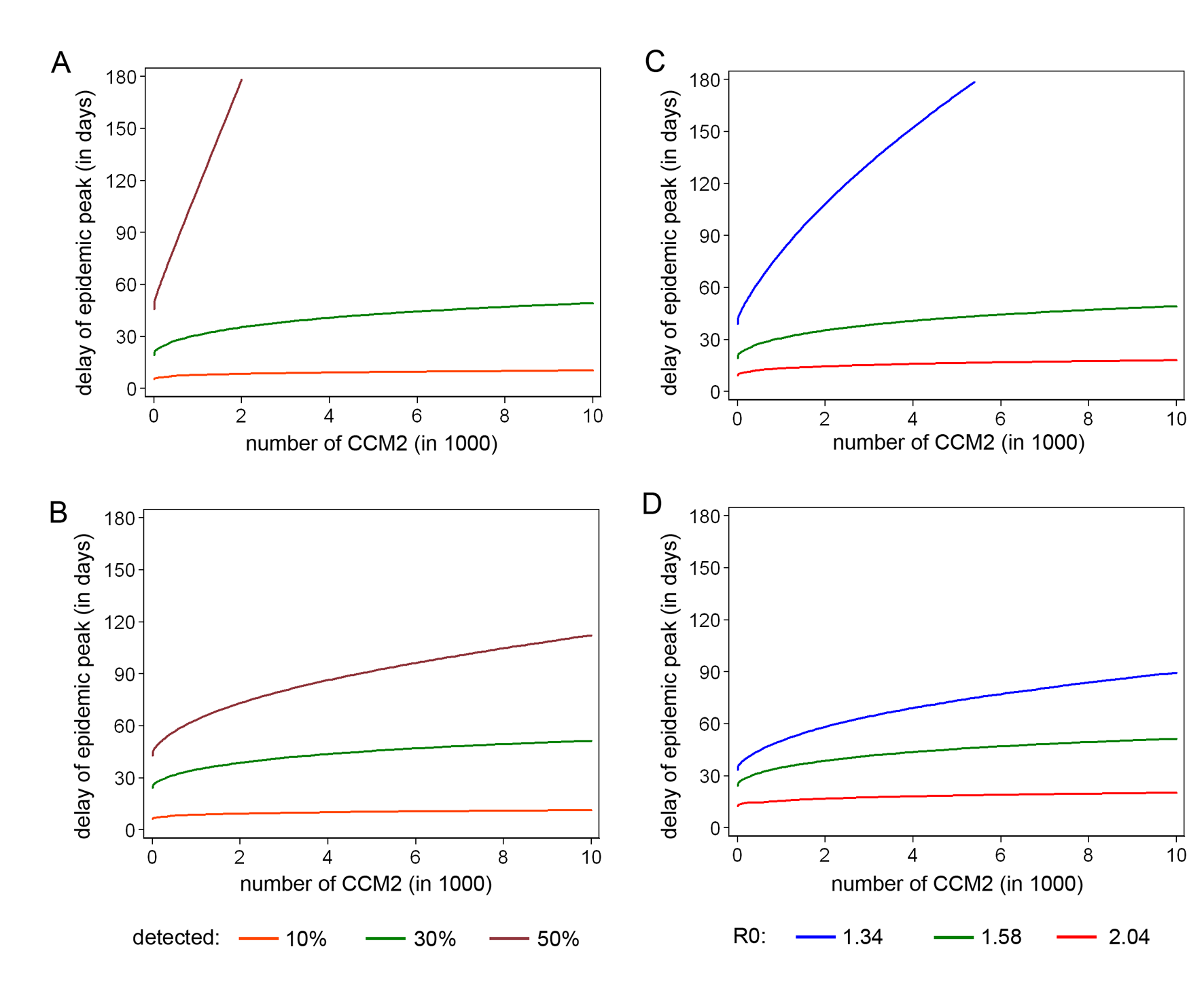

Supplement: Figure S4 — Delay of the peak of the epidemic depending on the number of CCM2 treatments. The respective curves start where CCM1 has taken effect already in the first 500 detected cases. (A and B): The delay in days is presented for case detection rates of 10%, 30%, and 50%. R0 is set to 1.58. (A) shows the delay when 5 cases are imported per day, and (B) shows when an exponentially increasing number of cases, but not more than 120, are imported per day. (C and D): The delay in days is presented for basic reproduction numbers of 1.34, 1.58, and 2.04. The case detection rate is set to 30%. (C) shows the delay when 5 cases are imported per day, and (D) shows when an exponentially increasing number of cases, but not more than 120, are imported per day. (7.72 MB TIF) [file pone.0008356.s004.tif]
